# *Cordyceps militaris* Solid Medium Extract Alleviates Lipoteichoic Acid-Induced MH-S Inflammation by Inhibiting TLR2/NF-κB/NLRP3 Pathways

**DOI:** 10.3390/ijms242115519

**Published:** 2023-10-24

**Authors:** Xiaoya Wang, Zhiping Peng, Lei Wang, Jingyan Zhang, Kang Zhang, Zhiting Guo, Guowei Xu, Jianxi Li

**Affiliations:** Engineering & Technology Research Center of Traditional Chinese Veterinary Medicine of Gansu Province, Lanzhou Institute of Husbandry and Pharmaceutical Sciences, Chinese Academy of Agricultural Sciences, Lanzhou 730000, China

**Keywords:** *Cordyceps militaris* solid medium, LTA, MH-S, inflammation

## Abstract

The aim of this study was to investigate the inhibitory effects of *Cordyceps militaris* solid medium extract (CME) and cordycepin (COR) on LTA-induced inflammation in MH-S cells and their mechanisms of action. In this study, the establishment of an LTA-induced MH-S inflammation model was determined, the CCK-8 method was used to determine the safe concentration range for a drug for COR and CME, the optimal concentration of COR and CME to exert anti-inflammatory effects was further selected, and the expression of inflammatory factors of TNF-α, IL-1β, IL-18, and IL-6 was detected using ELISA. The relative expression of TNF-α, IL-1β, IL-18, IL-6, IL-10, TLR2 and MyD88 mRNA was detected using RT-PCR, and the IL-1β, IL-18, TLR2, MyD88, NF-κB p-p65, NLRP3, pro-caspase-1, Caspase-1 and ASC protein expression in the cells were detected using Western blot; immunofluorescence assay detected the expression of Caspase-1 in MH-S cells. The results revealed that both CME and COR inhibited the levels of IL-1β, IL-18, IL-6, and TNF-α in the supernatants of LTA-induced MH-S cells and the mRNA expression levels of IL-1β, IL-18, IL-6, TNF-α, TLR2 and MyD88, down-regulated the LTA-induced IL-1β, IL-18, TLR2 in MH-S cells, MyD88, NF-κB p-p65/p65, NLRP3, ASC, pro-caspase-1, and caspase-1 protein expression levels, and inhibited LTA-induced caspase-1 activation in MH-S cells. In conclusion, CME can play a therapeutic role in LTA-induced inflammation in MH-S cells via TLR2/NF-κB/NLRP3, and may serve as a potential drug for bacterial pneumonia caused by Gram-positive bacteria.

## 1. Introduction

Gram-positive bacterial infections are one of the main causes of lung inflammation, the most common of which are *Streptococcus pneumoniae* and *Staphylococcus aureus* (*S. aureus*). Lipoteichoic acid (LTA) is a key component of Gram-positive bacteria [1]; belonging to the glycerophosphoric acid chain molecules covalently bonded with phospholipids on cell membranes to form a complex, LTA can be recognized by the TLR2 receptor [2], and can cause further inflammatory damage to the body through activation of the inflammatory pathway [3]. Macrophages are the first line of defense of natural immunity and initiate a transient immune response when subjected to pathogen-associated molecules (PAMP) or damage-associated (DAMP) molecular patterns during the detection of foreign pathogens [4,5]. Alveolar macrophages are the main immune effector cells within the alveolar structure, and they are the first immune cells to encounter inhaled pathogens (e.g., mosquitoes, environmental toxins, or allergens) [6], initiating a transient immune response when subjected to PAMP or DAMP molecular patterns [4]. Macrophages sense PAMP and DAMP through TOLL-like receptors (TLR) and NOD-like receptors (NLR) [7], and, unlike the TLR, the NLR can respond to its specific stimuli to form inflammasomes. The NOD-like receptor thermal protein domain-associated protein 3 (NLRP3) is one of the most characterized members of the NLR, and the NLRP3 inflammasome consists of NLRP3, an apoptosis-associated speck-like protein containing a CARD (ASC), and cystathionine-1 (Caspase-1), which is a multiprotein oligomer that activates the NF-κB signaling pathway to upregulate NLRP3 when mediated by microbial molecules or endogenous cytokines [8]. When mediated by DAMP, NLRP3 inflammatory vesicles activate and the protein structure opens up to recruit the downstream molecules ASC and pro-Caspase-1 to form the NLRP3-ASC-pro-Caspase-1 complex, prompting ASC to cleave pro-Caspase-1 into active Caspase-1 [9]. Caspase-1 then cleaves the cytokine pro-interleukinogen-1β (pro-IL-1β) and pro-IL-18 via protein hydrolysis, leading to the maturation and release of IL-1β and IL-18 [10]. Ginsenoside Rg1 [11], Baicalin [12], and Naringenin [13] have suppressed inflammatory expression by inhibiting the expression of proteins such as NF-κB and NLRP3.

**Figure 1:**
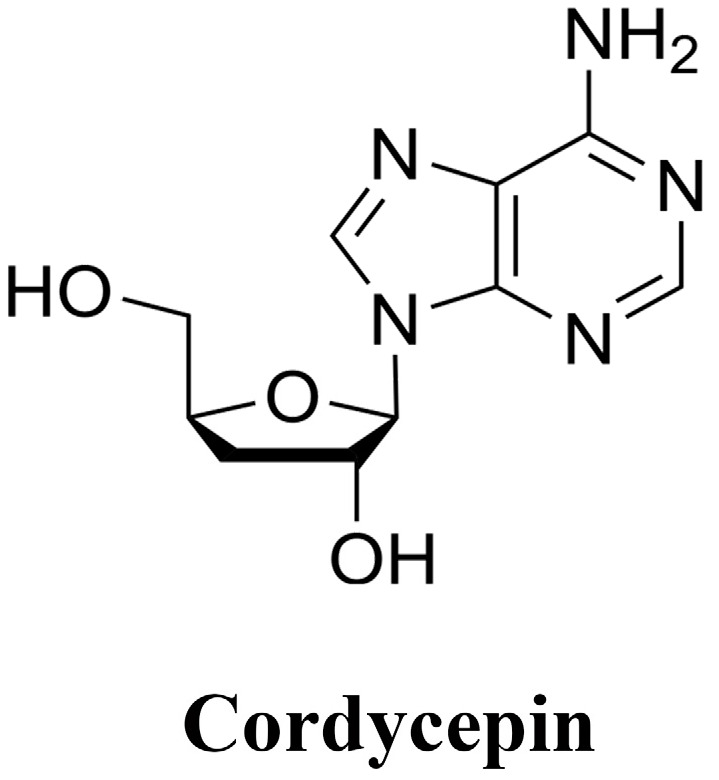
Chemical structure of Cordycepin.

*Cordyceps militaris*, a genus of Cordyceps in the family Ergotaceae, is a medicinal and food fungus of *Cordyceps militaris*, and it is well known that *Cordyceps militaris* fruiting bodies contain active ingredients such as cordycepin and adenosine [14]; the chemical structure is shown in Figure 1. According to the ancient books, *Cordyceps militaris* has the efficacy of relieving coughs, calming asthma, and clearing the lungs and resolving phlegm, and modern pharmacology has also proved that cordycepin was able to suppress the inflammation damage in lungs through the inhibition of various inflammatory pathways such as Nuclearfactor erythroidderived 2-like 2/heme oxygenase-1 (Nrf2/HO-1), NF-κB [15,16,17], and Amp-activated protein kinase (AMPK) [18], to inhibit inflammatory injury in the lungs and exert anti-inflammatory effects in macrophages [19]. The extract of *Cordyceps militaris* fruiting bodies also exhibits antioxidant [20], antimicrobial and anti-inflammatory effects [21,22], and is protective against membranous glomerulonephritis [23], asthma [24], colitis [25] and hyperlipidemia [26] in mice. However, due to the lack of wild resources of *Cordyceps militaris*, the technology of in vitro cultivation has also developed rapidly [27]. *Cordyceps militaris* solid medium is cultivated with wheat kernel, brown rice, soybeans, milk powder and other solid miscellaneous media to cultivate *Cordyceps militaris* fungus; *Cordyceps militaris* fungus is used as a tool for ecological transformation and for the process of successfully cultivating the substrate while obtaining the whole of the culture medium and the mycelium coexisting together. It is worth mentioning that when *Cordyceps militaris* fruiting body is harvested, a large amount of raw medium will be left over [28], resulting in a waste of resources. *Cordyceps militaris* solid medium is full of mycelium, which also possesses active ingredients such as cordycepin [29,30] and adenosine [31]. Actually, *cordyceps militaris* solid medium shows pharmacological effects, and Chen [32] has reported that *cordyceps militaris* solid medium can enhance the body’s immune function, promote the proliferation of beneficial bacteria, regulate immunity and promote intestinal health in mice. He found that *Cordyceps militaris* solid medium extract has antioxidant and antibacterial ability [33]. It is hypothesized that its extracts also have the same effect of inhibiting the damage caused by pneumonia, but whether the extracts of *Cordyceps militaris* solid medium are able to attenuate the inflammation of alveolar macrophage cells induced by LTA, and whether it exerts anti-inflammatory effects through the TLR2/NF-κB/NLRP3 pathway is not clear. Using cordycepin as a control, this experiment was designed to determine the protective effects and potential mechanisms of *Cordyceps militaris* solid medium extracts (CME) against LTA-induced inflammation in MH-S.

## 2. Results

### 2.1. Effect of LTA on Survival Rate of MH-S Cells

**Figure 2:**
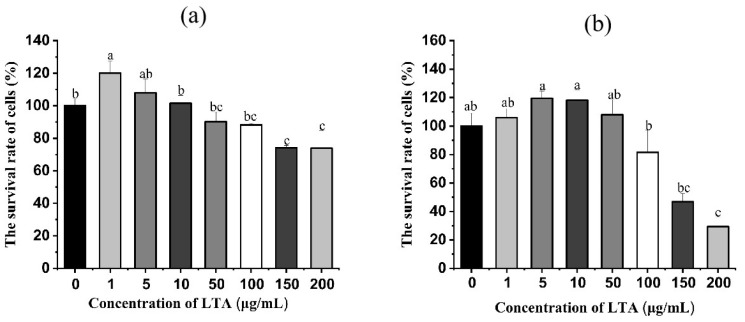
(**a**) Cell survival of MH-S cells after 12 h stimulation with different concentrations of LTA. (**b**) Cell survival after 24 h of LTA stimulation of MH-S cells at different concentrations. Different lowercase letters on the error bars indicate statistically significant differences (*p* < 0.05).

In order to ascertain the concentration and time of LTA, LTA was prepared in a different concentration and stimulated cells for 12 h and 24 h, respectively. Then the survival rate of MH-S cells was detected using the CCK-8 method. As shown in Figure 2, when LTA was stimulated for 12 h and 24 h, compared with the blank group, the cell survival rate of MH-S was not significantly affected when LTA was less than 100 ug/mL, while the cell survival rate of MH-S was significantly decreased when LTA concentration was 150 and 200 μg/mL (*p* < 0.05), Cell survival rates were 74.08% and 73.88% when the cell was stimulated by LTA for 12 h, and the survival rate was 46.85% and 29.34% when the cell was stimulated by LTA for 24 h. Thus, 200 ug/mL LTA was used to establish inflammation model.

### 2.2. Effect of LTA Stimulation on Protein Expression

**Figure 3:**
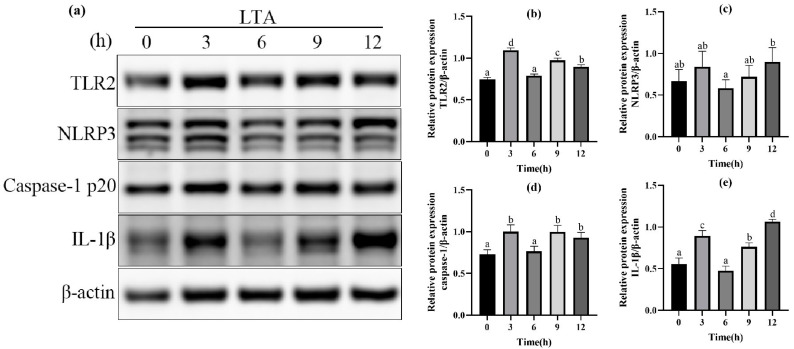
(**a**) Western blot images of TLR2, NLRP3, Caspase-1, IL-1β and β-actin in MH-S cells. (**b**) The relative protein expression of TLR2. (**c**) The relative protein expression of NLRP3. (**d**) The relative protein expression of Caspase-1. (**e**) The relative protein expression of IL-1β. Different lowercase letters above error bars indicate statistically significant differences (*p* < 0.05).

In order to further determine the optimal stimulation time of MH-S cells induced by LTA, different stimulation times (0, 3, 6, 9, 12 h) of LTA with the final concentration of 200 μg/mL were used. The protein expressions of TLR-2, NLRP3, Caspase-1 and IL-1β in MH-S cells were detected using Western blot, and are shown in Figure 3. The results showed that when LTA stimulated MH-S cells for 3 h and 12 h, compared with the blank group, the protein expression of TLR2, Caspase-1 and IL-1β was significantly up-regulated (*p* < 0.05); NLRP3 protein expression was up-regulated in MH-S cells stimulated by LTA for 3 h and 12 h, but the difference was not significant compared with the blank control group (*p* < 0.05). The protein expressions of TLR2 and Caspase-1 peaked at 3 h after LTA stimulation, and those of NLRP3 and IL-1β peaked at 12 h after LTA stimulation. In conclusion, we selected 200 μg/mL LTA to stimulate MH-S cells for 12 h to establish the cell inflammation model.

### 2.3. Effect of CME and COR on Survival Rate of MH-S Cells

**Figure 4:**
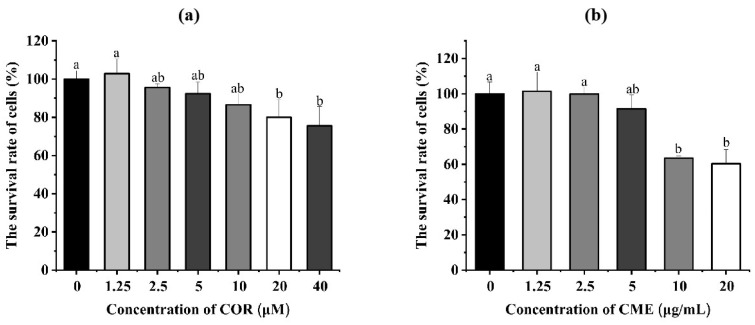
(**a**) Cell viability of MH-S cells treated with different concentrations of CME for 12 h. (**b**) Cell viability of MH-S cells treated with different concentrations of COR for 12 h; different lowercase letters above error bars indicate statistically significant differences (*p* < 0.05).

The different concentrations of COR and CME were applied, respectively, to MH-S cells for 12 h. Cell viability was determined using the CCK-8 assay. As Figure 4a shows, compared with the blank control group, when the CME was at 10, 50 and 100 μg/mL, the difference in the survival rate of MH-S cells was not significant (*p* > 0.05); when the concentration of CME was 500 and 1000 μg/mL, the cell survival rate was significantly reduced (*p* < 0.05), and the cell survival rate was 63.55% and 60.29%. Compared with the blank group, there was no significant decrease in cell survival when the COR concentration was less than or equal to 10 μM. At COR concentrations of 20 μM and 40 μM, the cell survival rates were 80.01% and 75.57%, which were significantly different from the blank group.

### 2.4. Screening for Optimal Concentration of CME and COR

**Figure 5:**
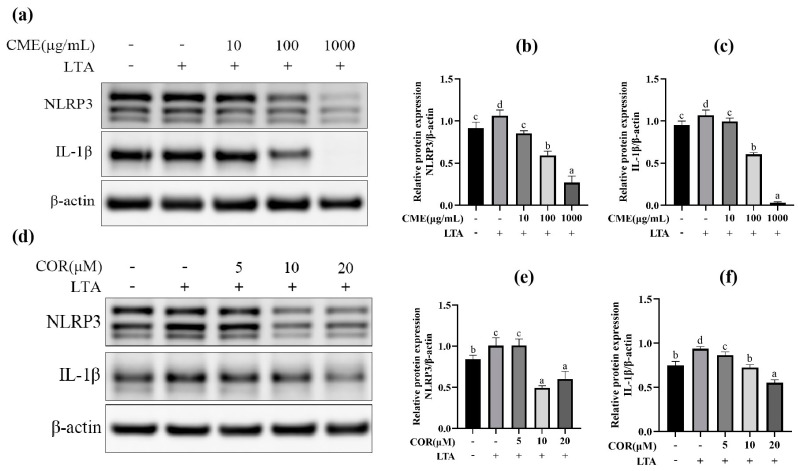
The effect on protein of different concentration of CME and COR. (**a**) Western blot band of CME; the relative expression of (**b**) NLRP3, (**c**) IL-1β, (**d**) Western blot band of COR. The relative expression of (**e**) NLRP3, (**f**) IL-1β. Different lowercase letters above error bars indicate statistically significant differences (*p* < 0.05).

At first, the three different concentrations of CME and COR were applied to the MH-S cells for 3 h and then stimulated with LTA (200 μg/mL) for 12 h. Then, the optimal concentrations of CME and COR were selected by detecting the expression of TLR-2, NLRP3, Caspase-1 and IL-1β. The results are shown in Figure 5. Pretreatment of MH-S cells with different concentrations of CME significantly reduced NLRP3 and IL-1β protein expression in the cells in a dose-dependent manner (*p* < 0.05). The results indicated that CME reduced the secretion of NLRP3 and IL-1β in LTA-induced MH-S cells, and the final concentration of CME was determined as 100 μg/mL.

We tried to select the optimal concentration from 5, 10, and 20 μM COR, NLRP3. The IL-1β protein expression was significantly elevated after LTA stimulation of MH-S cells: when the COR concentration was 10 μM and 20 μM, it significantly reduced the expression of NLRP3 and IL-1β proteins in LTA-induced MH-S cells, and 20 μM of COR was chosen as the optimal concentration.

### 2.5. The Effect of CME and COR on Secretion of Inflammation Factors in LTA-Induced MH-S Cells

**Figure 6:**
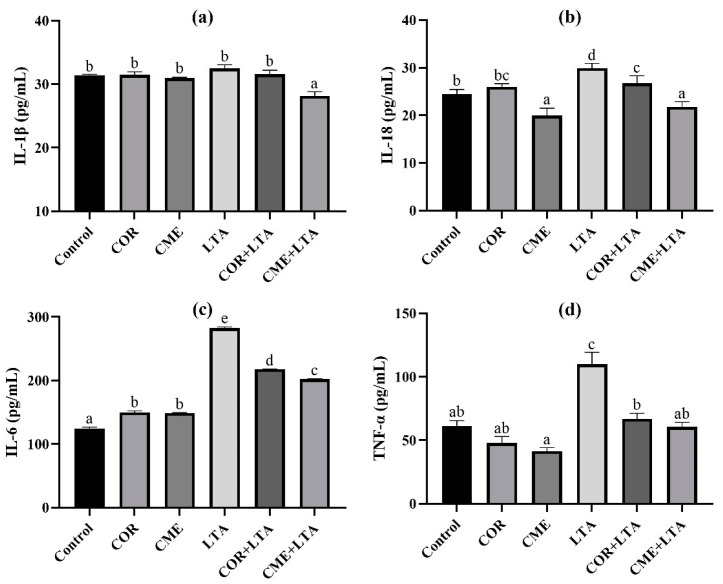
Effects of CME on content of inflammatory cytokines of supernatant in LTA-induced MH-S cell. The content of (**a**) IL-1β, (**b**) IL-18, (**c**) IL-6 and (**d**) TNF-α in each group. Different lowercase letters above error bars indicate statistically significant differences (*p* < 0.05).

ELISA was used to detect the secretion of inflammatory cytokines IL-1β, IL-18, IL-6 and TNF-α in MH-S cells stimulated by LTA for 12 h after pretreatment with CME and COR for 3 h. As can be seen from Figure 6, compared with the blank control group, the effects of CME and COR pretreatment on MH-S were observed, and the contents of IL-1β and TNF-α in the cell supernatant were not significantly different (*p* > 0.05); the content of IL-6 in the cell supernatant was significantly increased (*p* < 0.05), there was no significant difference in IL-18 content in the cell supernatant after COR pretreatment (*p* > 0.05), and the content of IL-18 in the cell supernatant was significantly decreased after CME pretreatment (*p* < 0.05). After LTA stimulated MH-S cells for 12 h, the contents of IL-18, IL-6 and TNF-α in the cell supernatant were significantly higher than those in the blank (*p* < 0.05), although the IL-1β content showed no significant difference (*p* < 0.05). CME and COR pretreatment significantly decreased the contents of IL-18, IL-6 and TNF-α in the LTA-induced MH-S cell supernatant (*p* < 0.05), and the content of TNF-α was not significantly different from that of the blank group (*p* < 0.05). In addition, the reduction in IL-18 and IL-6 inflammatory factors by CME was significantly greater than that by COR.

### 2.6. CME and COR Inhibit the Relative mRNA Expression of Inflammatory Factor in LTA-Induced MH-S Cells

**Figure 7:**
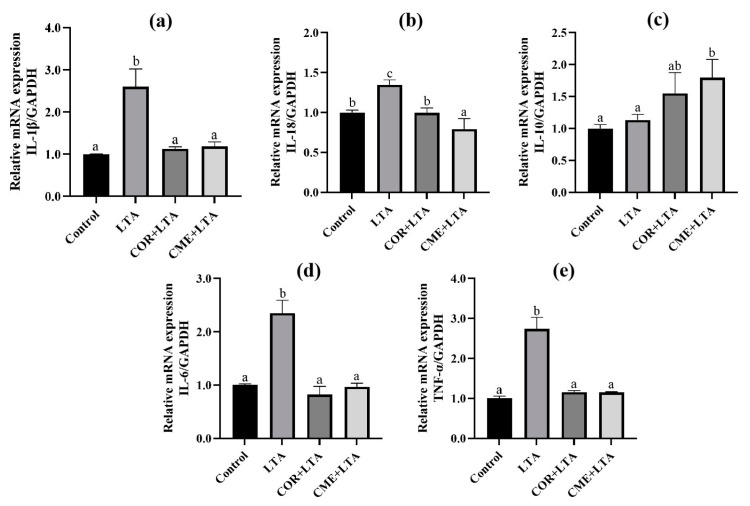
Effects of COR and CME on the mRNA expression of related inflammatory cytokines in LTA-induced MH-S cells. The relative mRNA expressions of (**a**) IL-1β, (**b**) IL-18, (**c**) IL-10, (**d**) IL-6 and (**e**) TNF-α in each group of MH-S cells. Different lowercase letters above error bars indicate statistically significant differences (*p* < 0.05).

RT-PCR was used to detect the effects of COR and CME on MH-S cells, and the mRNA expression of inflammatory factors (IL-1β, IL-18, IL-10, IL-6 and TNF-α) in the supernatant of MH-S cells stimulated by LTA. Results as shown in Figure 7 show that, compared with the blank control group, LTA significantly stimulated the mRNA expression of IL-1β, IL-18, IL-6 and TNF-α in cells (*p* < 0.05). Compared with the LTA model group, COR and CME pretreatment significantly decreased the mRNA expression of IL-1β, IL-18, IL-6 and TNF-α in cells (*p* < 0.05), and the mRNA expression levels of IL-1β, IL-6 and TNF-α were not significantly different from those of the blank group (*p* > 0.05). There was no significant difference between CME and COR pretreatment on the inhibition of MH-S cell inflammatory factors induced by LTA (*p* > 0.05).

### 2.7. CME Inhibited LTA-Induced Inflammation in MH-S Cells through TLR2/NF-κB Signal Pathway

**Figure 8:**
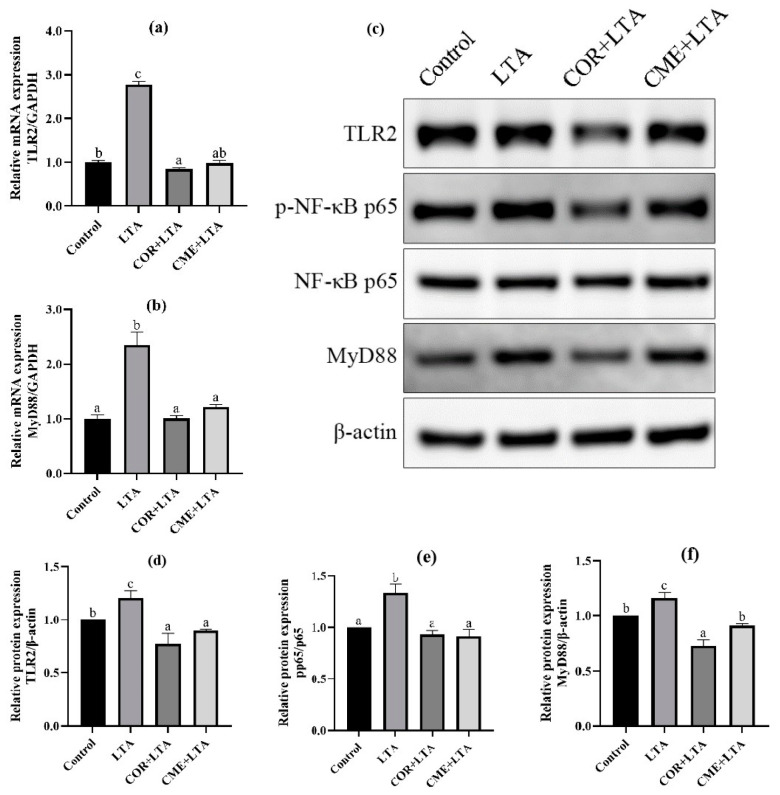
Effects of CME in LTA-induced MH-S cell inflammation model by TLR2/NF-κB. The relative mRNA expressions of (**a**) TLR2, (**b**) MyD88. (**c**) WB blot images. The relative expression of protein (**d**) TLR2/β-actin, (**e**) MyD88/β-actin, (**f**)p-p65/p65. Note: Value columns with different lowercase letters mean significant difference (*p* < 0.05).

In order to explore whether CME inhibits inflammation through the TLR2/NF-κB signaling pathway, RT-PCR was used to detect the mRNA expression levels of TLR2 and myelinated primary response 88 (MyD88). It can be seen from Figure 8 that, compared with the blank control group, LTA stimulation can significantly up-regulate the mRNA expression of TLR2 and MyD88 in MH-S cells (*p* < 0.05). CME and COR pretreatment of MH-S cells significantly decreased the mRNA expression of TLR2 and MyD88 in LTA-induced cells (*p* < 0.05), and CME reduced the mRNA expression of TLR2 and MyD88 in cells to a normal level without significant difference (*p* > 0.05). In addition, there was no significant difference in TLR2 mRNA expression level between the COR group and blank control group (*p* > 0.05), and MyD88 mRNA expression level was even significantly lower than that of the blank group (*p* < 0.05). CME showed no significant difference in inhibitory ability from COR (*p* > 0.05).

WB detection was used to further determine whether CME and COR pretreatment could affect the expression of TLR2/NF-κB signaling pathway-related proteins to inhibit cell inflammatory damage. The Western blot banding can be seen in Figure 8c. The protein expression levels of the TLR2 receptor and MyD88 increased significantly after LTA stimulation, and the p-p65/p65 ratio increased significantly, indicating that LTA activated the expression of TLR2 and MyD88 proteins, increased the phosphorylation of p65, and was subsequently transferred to the nucleus [34,35], thereby causing inflammation in the MH-S cells. Not surprisingly, COR and CME significantly decreased the expression of TLR2 and MyD88 proteins (*p* < 0.05), and then inhibited the phosphorylation of p65 so that the p-p65/p65 ratio would reach the normal level, and there was no significant difference from the normal control group (*p* > 0.05). The results show that CME can reduce inflammatory damage by inhibiting the activation of TLR2 and NF-κB signaling pathways. In addition, the anti-inflammatory capacity of CME was better than COR, but not significantly. 

### 2.8. CME Inhibited LTA-Induced MH-S Cell Inflammatory Response through NLRP3

**Figure 9:**
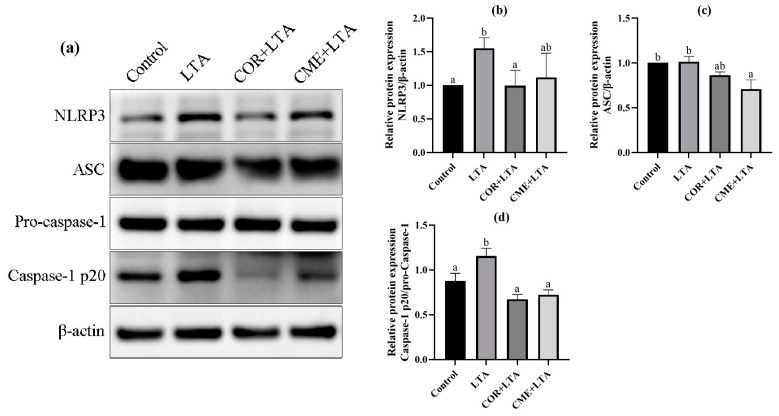
Effect of CME on LTA-induced NLRP3 inflammation-associated proteins in MH-S cell. (**a**) Protein immunoblotting bands of NLRP3, ASC, pro-Caspase-1, Caspase-1 and β-actin in MH-S cells. The relative expression of protein (**b**) NLRP3/β-actin, (**c**) ASC/β-actin, (**d**) Caspase-1 p20/pro-Caspase-1. Note: different lowercase letters on the error bars indicate statistically significant differences (*p* < 0.05).

In order to investigate whether CME alleviates LTA-induced inflammatory damage in MH-S cells by inhibiting the NLRP3 inflammasome complex, the protein expressions of NLRP3, ASC and Caspase-1 in MH-S cells were detected using the WB assay. As shown in Figure 9, LTA stimulation of MH-S cells significantly upregulated the NLRP3 protein (*p* < 0.05) and promoted the expression of the ASC protein (*p* > 0.05). The Caspase-1 p20/Pro-Caspase-1 ratio was significantly increased (*p* < 0.05), activating the NLRP3/ASC pathway. CME inhibited the expression of the NLRP3 protein induced by LTA and significantly decreased the expression of the ASC protein (*p* < 0.05). The Caspase-1 p20/Pro-Caspase-1 ratio was significantly reduced to that of the blank control group (*p* < 0.05); COR reduced NLRP3 protein expression and the Caspase-1 p20/Pro-Caspase-1 ratio in MH-S cells induced by LTA was reduced to a normal level, showing no significant difference compared with the blank control group (*p* > 0.05). The results showed that both COR and CME inhibited the activation of Caspase-1 and interfered with the function of NLRP3 inflammasome.

### 2.9. Effect of CME on Caspase-1 Activation of MH-S Cells Induced by LTA

**Figure 10:**
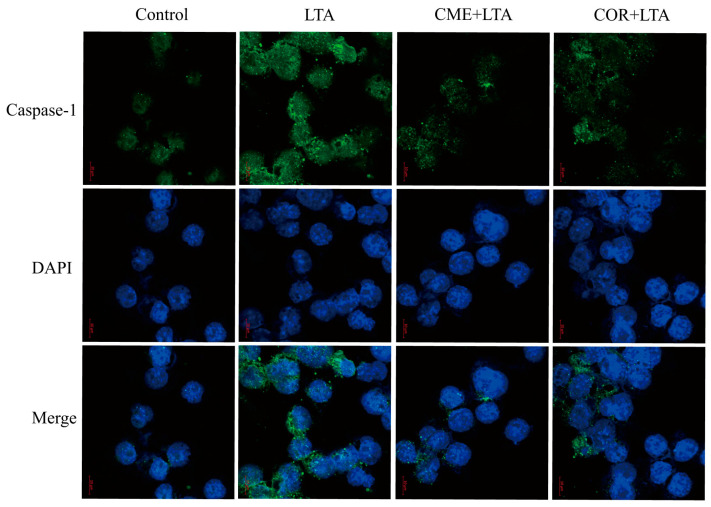
Effect of CME and COR on the activation of Caspase-1 in LTA-induced MH-S cells.

In order to further demonstrate the effect of CME on LTA-induced Caspase-1 activation of MH-S cells, the expression of Caspase-1 in the blank control group, the LTA model group and MH-S cells pretreated with CME and COR for 3 h was detected using the immunofluorescence assay. As shown in Figure 10, compared with the blank control group, LTA significantly induced the formation of perinuclear fluorescence spots of Caspase-1 in MH-S cells. Compared with the LTA group, both CME and COR can reduce the formation of perinuclear fluorescent spots. In conclusion, CME and COR can inhibit the Caspase-1 activation of MH-S cells induced by LTA, which is consistent with the WB results.

## 3. Discussion

*S. aureus* is a Gram-positive bacterium that is the main pathogen of pneumonia infections [36]. LTA, a surface-associated adhesion amphiphile from Gram-negative bacteria and a regulator of autolytic wall enzymes (cytosolic wall enzymes), which is released from bacterial cells mainly after lysozyme, leukocyte cationic peptide, or β-lactam antibiotic-induced lysis, is an important constituent of the pathogen-associated molecular pattern PAMPs, which leads to the activation of NF-κB. NF-κB drives the expression of target genes that mediate cell proliferation, as well as the release of antimicrobial molecules and cytokines to activate the immune response [37]. LTA can be considered a virulence factor [38] and is a major driver of the host’s inflammatory response to such bacteria [39]. It binds non-specifically to membrane phospholipids with target cells or specifically to Toll-like receptors; LTA binding to targets can be inhibited by antibodies, phospholipids, and antibodies specific for CD14 and Toll. The primary receptor recognizing LTA is TLR2, which is initiated by the internalization of TLR2, and TLR-signaling junction proteins, MyD88, are recruited to the cell membrane which transduces NF-κB-dependent inflammatory signaling [40].

It was found that acute lung injury was induced by intratracheal injection of LTA in mice, and that LTA induced the activation of lung epithelial cells, lung endothelial cells, lung mesenchymal stromal cells, alveolar macrophages, and the production of a large number of inflammatory mediators (IL-6, IL-1β, TNF-α, and KC) and oxygen free radicals (NO), which are involved in the recruitment of other immune cells (e.g., eosinophils or neutrophils). These were induced into the lung, limiting lung inflammation [41]. Alveolar macrophage phagocytose and clear foreign bodies activate immune responses, induce inflammatory responses, and play a key role in immune defense (MAN et al., 2017). In this study, we found that stimulation of MH-S cells with 200 μg/mL of LTA for 12 h resulted in a significant decrease in cell viability and elevated the expression of TLR2, NLRP3, Caspase-1 and IL-1β proteins in the cells. Therefore, 200 μg/mL LTA was finally chosen to stimulate MH-S cells for 12 h to establish the inflammation model.

*Cordyceps militaris* is a medicinal fungus that has long been used traditionally in East Asia, including China, for a variety of medical purposes [42]. For example, it has been found that *Cordyceps militaris* extracts and its active ingredients inhibit the expression of inflammatory mediators (IL-1β, IL-6, IL-18, TNF-α, NO) which may be regulated via signaling pathways such as TLR2, NF-κB, MAPK, and others [21,43,44]. However, large-scale cultivation of *Cordyceps militaris* is not only labor-intensive, but also leaves a large amount of discarded solid culture-medium residue of the body of *Cordyceps militaris* after the substrate is harvested. The culture residue of *Cordyceps militaris* contains a large number of mycelium, which has similar active components to the substrate [45] (cordycepin) and possesses antimicrobial, anti-inflammatory and antitumor activities. At present, there are only a few studies on *Cordyceps militaris* solid medium. In this study, we found that there was no significant change in cell viability after co-culturing low concentrations of CME (10, 50, 100 μg/mL) and COR (1.25, 2.5, 5, 10 μM) with MH-S cells for 12 h. At the same time, the expression of relevant proteins in the LTA-induced MH-S cells were examined using Western blot after pretreatment with CME and COR, and the results showed that CME and COR could inhibit the elevated protein expression of NLRP3 and IL-1β in LTA-induced MH-S cells. Therefore, we selected the optimal CME concentration of 100 μg/mL and COR concentration of 20 μM from these, and treated the cells for 3 h to further investigate whether CME and COR could attenuate LTA-induced inflammatory injury in MH-S cells.

Inflammatory factors play an important role in acute inflammatory injury. It was demonstrated that *S. aureus* LTA induced a macrophage inflammatory response, which was closely related to the secretion of inflammatory factors (IL-1β, IL-6, IL-18, TNF-α). The ELISA, RT-PCR and Western blot assays in this study revealed that after stimulation of MH-S cells with 200 μg/mL of LTA for 12 h, the secretion of the inflammatory factors IL-6, IL-18, and TNF-α in the supernatants of the cells was increased, and the mRNA expression of IL-1β, IL-6, IL-18, and TNF-α in the cells was elevated. The mRNA expression of IL-1β, IL-6, IL-18 and TNF-α was elevated in the cells, and the protein expression of IL-1β and IL-18 was also elevated in the cells. The results indicated that 200 μg/mL of LTA induced an inflammatory response in MH-S cells. In addition, this study found that both CME and COR pretreatment significantly inhibited the LTA-induced increase in the secretion of inflammatory factors IL-6, IL-18, and TNF-α in the supernatants of the MH-S cells, while significantly increasing the mRNA expression of the inflammation-suppressing factor IL-10. In addition, both CME and COR pretreatment significantly inhibited the mRNA expression of IL-1β, IL-6, IL-18 and TNF-α in LTA-induced MH-S cells, while significantly suppressing IL-1β and IL-18 protein expression in the cells. The results indicated that both CME and COR could attenuate the inflammatory response induced by LTA in MH-S cells and significantly inhibit the expression of inflammatory factors IL-1β, IL-6, IL-18 and TNF-α, and that both had similar anti-inflammatory effects.

IL-1β is one of the important pro-inflammatory cytokines involved in innate immunity, and it requires two signals for activation: the first signal usually comes from the activation of TLRs, which promotes the expression of pro-IL-1β, and the second signal is the activation of inflammatory vesicle-dependent caspase-1, which promotes the conversion of pro-IL-1β to mature IL-1β [46]. The effect of CME on LTA-induced cells and the mechanism of inflammatory response is not clear, so this study investigated whether the anti-inflammatory effect of CME and COR is related to the TLR2/NF-κB/NLRP3 inflammation signaling pathway. It was demonstrated that LTA can be recognized by TLR2 on the cell membrane, and this activates the innate immunity of the organism [47]. TLR2 is a plasma membrane-bound pattern recognition receptor that recognizes bacterial lipoproteins, lipopeptides, and lipophospholipids, and signals a heterodimer with either TLR1 or TLR6. The TLR2:TLR6 heterodimer specifically recognizes diacyl lipoproteins of Gram-positive bacteria; the TLR2:TLR1 heterodimer recognizes triacyl lipoproteins of Gram-negative bacteria [48]. The classical TLR2 signaling pathway works by forming a heterodimer with TLR1 or TLR6, recruiting TIRAP and MyD88 proteins, forming a Myddosome complex at the cell membrane, and inducing pro-inflammatory cytokine secretion with dependence on NF-κB [49]. The results of RT-qPCR and Western blot in this study showed that both CME and COR inhibited TLR2 and MyD88 mRNA and protein expression and suppressed phosphorylation of the NF-κB p65 protein in *S. aureus* LTA-induced MH-S cells, suggesting that CME and COR may attenuate LTA-induced inflammatory responses via the TLR2 signaling pathway.

Inflammasomes are important members of the innate immune response, of which the most studied is NLRP3 [7]. When NLRP3 inflammasome is activated, NLRP3, ASC, and pro-Caspase-1 bind to each other; upon activation of the NLRP3 inflammasome complex, pro-Caspase-1 undergoes auto-cleavage into active Caspase-1 p10/p20 and Caspase-1 aggregates and then activates and induces IL-1β and IL- 18 secretion [50]. Studies have reported that LTA is recognized by TLR2, activates NF-κB, and induces the transcriptional expression of pro-IL-1β and NLRP3 inflammatory vesicles. Yang et al. [51] found that COR pretreatment inhibited LPS-induced inflammatory mediator (IL-1β, IL-6, TNF-α, MCP-1 and COX-2) expression and was found to exert anti-inflammatory effects via ERK1/2 and NLRP3 inflammasome signaling pathways. To demonstrate whether the LTA-induced activation of IL-1β and IL18 in this study was dependent on NLRP3 inflammatory vesicles, it was found that stimulation of MH-S cells with 200 μg/mL of LTA for 12 h significantly increased the expression of NLRP3 and Caspase-1 p20 in the cells, whereas there was no significant change in the expression of ASC in the cells. In addition, both CME and COR down-regulated the LTA-induced elevation of IL-1β and IL-18 expression, while CME and COR pretreatment significantly inhibited LTA-induced NLRP3 and Caspase-1 p20 expression in MH-S cells without significant changes in ASC expression in the cells, in which the two inhibitory effects were similar. CME may inhibit NLRP3 and Caspase-1 p20 expression in MH-S cells by disrupting the pro-Caspase-1 self-cleavage, reduce the activation of Caspase-1, and thus reduce the secretion of inflammatory factors. This needs to be continued in order to study the specific location of its target of action in depth later.

## 4. Materials and Methods

### 4.1. Reagent

*Cordyceps militaris* solid medium was purchased from Shandong Ogilvy Biological Engineering Co. Ltd. (Shangdong, China); mouse alveolar macrophage line MH-S cells were purchased from Wuhan Punosai Biotechnology Co., Ltd. (Wuhan, China). *S. aureus* LTA was purchased from Sigma (Shanghai, China). Fetal bovine serum was purchased from Gibco (Grand Island, NE, USA). RPMI 1640 culture medium was purchased from Shanghai Yuanpei Biotechnology Co., Ltd. (Shanghai, China). CCK-8 reagent was purchased from ZETA Life (Beijing, China). Mouse IL-1β, IL-6, IL-18 and TNF-α ELISA kits were purchased from Shanghai Jianglai Biotechnology Co., Ltd. (Shanghai, China). IL-1β, IL-18, Caspase-1 p20/p10 and β-actin antibodies were purchased from Wuhan Sanying Biotechnology Co., Ltd. (Wuhan, China). MyD88, ASC, TLR2, NF-κB p65, and p-p65 antibodies were purchased from Cell Signaling Technology (Danvers, MA, USA). NLRP3 antibody was purchased from Thermo Fisher Technology Co., Ltd. (Shanghai, China).

### 4.2. The Methods of Extraction

According to the method of reference [52], *Cordyceps militaris* solid medium extract was obtained. The conditions are as follows: water at a solid–liquid ratio of 1:15, and then water reflux (at 70 °C for 60 min). Before being filtered, the COR content of extraction was 1.48 ± 0.52 mg/g. After freeze-drying, 200 mg water extraction of *Cordyceps militaris* solid medium was accurately weighed in a flask, dissolved in an appropriate amount of PBS, then placed in a 10 mL volumetric bottle. Ultrasound was applied for 30 min, it was shaken well at a constant volume, centrifuged at 3000 rpm for 10 min, and then the supernatant was filtered using a 0.45μm filter membrane. The final concentration was 20 mg/mL. Then it was filtered using a 0.22 μm filter membrane and stored in refrigerator at 4 °C.

### 4.3. Cell Culture

The complete medium was composed of 1640 medium containing 10% fetal bovine serum, 100 U/mL penicillin and 100 μg/mL streptomycin, which was stored at 4 °C for the reserve [53]. The cells were cultured in a cell culture at 37 °C and 5% CO_2_, and passage was carried out when the cells grew by more than 80%.

### 4.4. LTA Stimulates MH-S Cell Inflammation

MH-S cells at logarithmic growth stage were inoculated on 96-well plates and 6-well plates. When the cell fusion degree reached 80%, the MH-S cells were treated with 0, 1, 5, 10, 50, 100, 150, and 200 μg/mL LTA, respectively, and stimulated for 12 h and 24 h. The survival rate of the MH-S cells was determined using the CCK-8 method. The molding concentration of the LTA was determined. A total of 200 μg/mL LTA was added to stimulate the MH-S cells for 3 h, 6 h, 9 h and 12 h, respectively. Western blot was used to detect the expression of TLR2, NLRP3, Caspase-1 and IL-1β, and the stimulation time of the LTA was determined.

### 4.5. Screening for Optimal Concentration of COR and CME

The density of the MH-S cells was adjusted to 5 × 10^5^ cells/mL, and the cells were inoculated in 96-well plates and cultured at 37 °C and 5% CO_2_ for 24 h; then the medium was discarded. The MH-S cells were stimulated for 12 h by adding COR at the final concentration of 0, 1.25, 2.5, 5, 10, 20, and 40 μM, and CME at the final concentration of 0, 10, 50, 100, 500, and 1000 μg/mL, respectively. The safe concentration of cordycepin and CME on MH-2 cells was determined using CCK-8. A total of 10 μL CCK-8 solution was added to each group, and the absorbance (OD) was measured at 450 nm after 30 min in the incubator [54]. Then, the three different concentrations of COR and CME were selected by detecting the expression ability of NLRP3 and IL-1β, respectively.

### 4.6. Experimental Grouping and Drug Treatment

To detect the effects of COR and CME on TLR2/MyD88-related proteins in LTA-induced MH-S cells. The cell experiment was divided into four groups: the control group (Control), LTA group (LTA), COR+LTA group (COR+LTA) and CME+LTA group (CME+LTA) were treated with 20 μM cordycepin and 100 μg/mL CME, respectively, for 3 h, and then stimulated with 200 μg/mL LTA for 12 h.

### 4.7. ELISA Assay

MH-S cells at logarithmic growth stage were inoculated into 6-well plates, and the cells were treated using cell groups. The supernatant was collected from each group and centrifuged at room temperature for 10 min at 2000 r/min. The contents of TNF-α, IL-1β, IL-18 and IL-6 were determined using the ELISA kit [55].

### 4.8. ELISA Detection of Relative mRNA Expression by Real-Time PCR

In order to detect the expression of mRNA, total RNA was extracted from MH-S cells of each group using reagent and transcribed into cDNA, according to the manufacturer’s instructions. RT-PCR was performed on a real-time system spiked with SYBR Green to detect mRNA levels of IL-1β, IL-6, IL-10, IL-18, TNF-α, MyD88, and TLR2. GAPDH was used as the internal reference gene to determine the relative expression levels of the target genes, and the specific primers of each gene are listed in Table 1. Three biological replicates and three technical replicates were performed in all experiments. The relative mRNA expression was calculated using 2^−ΔΔCt^ [56].

### 4.9. WB Assay

Total protein was extracted from samples of each cell group, and the protein concentration of the samples was determined using the BCA kit. Sterile PBS solution was used to adjust the concentration to the same for each sample, protein loading buffer was added, and the sample was mixed and heated in a metal bath at 100 °C for 10 min. After cooling to room temperature, the samples were stored in a refrigerator at −20 °C. Samples were loaded according to 20~40 μg protein, electrophoresis was applied at constant pressure of 150 V for 50 min, and the samples were transferred to a PVDF membrane for 1 h under 200 mA; 5% skimmed milk powder was incubated at room temperature for 1 h, and the TBST was washed 3 times. The primary antibody was incubated at 4 °C overnight, the secondary antibody was incubated at room temperature for 1~2 h, and the TBST was washed 3 times. Finally, images were visualized using a luminescence imaging system, and the gray values of the strips were analyzed using ImageJ software version 1.53t.

### 4.10. Statistical Analysis

All the experiments were repeated three times; SPSS 26.0 was used to analyze the data and a one-way ANOVA (LSD) was applied. The results were expressed as the mean ± standard deviations and the difference was tested as the mean of multiple groups. Figures were created using Graphpad Prism 8.

## 5. Conclusions

Both the *Cordyceps militaris* solid medium extract and cordycepin inhibited the inflammatory response on LTA-induced MH-S by inhibiting the secretion of inflammatory factors (IL-1β, IL-18, TNF-α, and IL-6), and the mechanism of CME and COR on LTA-induced MH-S may be related to the inhibition of the TLR2/NF-kB signaling pathway and NLRP3 inflammasome activation. The results of this study suggest that CME may be a potential drug for the treatment of acute lung injury and lung inflammation, providing a theoretical basis for the development and utilization of *Cordyceps militaris* solid medium.

## Figures and Tables

**Table 1:** Sequence of primes for RT-PCR.

Gene	Primer Sequences (5′-3′)
GAPDH	Forward: TCATGAAACAAATGCATCTGG
	Reverse: TCATGAAACAAATGCATCTGG
IL-1β	Forward: GCAGGCAGTATCACTCATTGT
	Reverse: GGCTTTTTTGTTGTTCATCTC
IL-6	Forward: GTTCTCTGGGAAATCGTGGA
	Reverse: GCATTGGAAATTGGGCTAGG
IL-10	Forward: ACCTGGTAGAAGTGATGCCCCAGGCA
	Reverse: CTATGCAGTTGATGAAGATGTCAAA
IL-18	Forward: GCAGTAATACGGAGCATAAA
	Reverse: ATCCTTCACAGATAGGGTCA
TNF-α	Forward: AAGGGAGAGTGGTCAGGTTGG
	Reverse: CAGAGGTTCAGTGATGTAGCG
TLR2	Forward: ACGTTGGATGCCAAGTGCTGGG
	Reverse: ACGTTGGATCCACCCTGAA
MyD88	Forward: CGGAACTTTTCGATGCCTTTAT
	Reverse: CACACACAACTTAAGCCGATAG

## Data Availability

Not Applicable.

## Abbreviations

CME	*Cordyceps militaris* solid medium extract
COR	Cordycepin
*S. aureus*	*Staphylococcus aureus*
LTA	Lipoteichoic acid
PAMP	Pathogen-associated molecules
DAMP	Damage-associated
TLR	Toll-like receptors
NLR	NOD-like receptors
ASC	Apoptosis-associated speck-like protein containing a CARD
pro-IL-1β	Pro-interleukinogen-1β
Nrf2	Nuclearfactor erythroidderived 2-like 2
HO-1.	Heme oxygenase-1
AMPK	Amp-activated protein kinase
